# Carbon Monoxide Releasing Molecule-3 Enhances Heme Oxygenase-1 Induction via ROS-Dependent FoxO1 and Nrf2 in Brain Astrocytes

**DOI:** 10.1155/2021/5521196

**Published:** 2021-06-12

**Authors:** Chih-Chung Lin, Chien-Chung Yang, Li-Der Hsiao, Chuen-Mao Yang

**Affiliations:** ^1^Department of Anesthetics, Chang Gung Memorial Hospital at Linkou, and College of Medicine, Chang Gung University, Tao-Yuan 33302, Taiwan; ^2^Department of Traditional Chinese Medicine, Chang Gung Memorial Hospital at Tao-Yuan, Kwei-San, Tao-Yuan 33302, Taiwan; ^3^School of Traditional Chinese Medicine, College of Medicine, Chang Gung University, Kwei-San, Tao-Yuan 33302, Taiwan; ^4^Department of Pharmacology, College of Medicine, China Medical University, Taichung 40402, Taiwan; ^5^Ph.D. Program for Biotech Pharmaceutical Industry, China Medical University, Taichung 40402, Taiwan; ^6^Department of Post-Baccalaureate Veterinary Medicine, College of Medical and Health Science, Asia University, Wufeng, Taichung 41354, Taiwan

## Abstract

Carbon monoxide releasing molecule-3 **(**CORM-3) has been shown to protect inflammatory diseases via the upregulation of heme oxygenases-1 (HO-1). However, in rat brain astrocytes (RBA-1), the mechanisms underlying CORM-3-induced HO-1 remain poorly defined. This study used western blot, real-time PCR, and promoter activity assays to determine the levels of HO-1 expression and 2′,7′-dichlorodihydrofluorescein diacetate (H_2_DCFDA) and dihydroethidium (DHE) to measure reactive oxygen species (ROS). We found that CORM-3-induced HO-1 expression was mediated through ROS generation by Nox or mitochondria. The signaling components were differentiated by pharmacological inhibitors and small interfering RNA (siRNA). Subcellular fractions, immunofluorescent staining, and chromatin immunoprecipitation assay were used to evaluate the nuclear translocation and promoter binding activity of Nrf2 induced by CORM-3. The roles of mTOR and FoxO1 in CORM-3-stimulated responses are still unknown in RBA-1 cells. Our results demonstrated that transfection with siRNAs or pretreatment with pharmacological inhibitors attenuated the levels of HO-1 and phosphorylation of signaling components including Akt, mTOR, FoxO1, and Nrf2 stimulated by CORM-3. Moreover, pretreatment with N-acetyl-L-cysteine, diphenyleneiodonium chloride, apocynin, or rotenone blocked nuclear translocation and promoter binding activity of Nrf2 induced by CORM-3. The present study concluded that in RBA-1 cells, CORM-3-induced HO-1 expression is, at least partially, mediated through Nox and mitochondria/ROS-dependent PI3K/Akt/mTOR cascade to activate FoxO1 or ROS leading to activation of Nrf2 activity.

## 1. Introduction

Heme oxygenases (HOs) catabolize rate-limiting enzymatic degradation of heme into three products: free iron which triggers the induction of ferritin, biliverdin which is converted to bilirubin by biliverdin reductase, and carbon monoxide (CO). Three isoforms of HO have been characterized, including HO-1, HO-2, and HO-3 [1]. Among this family, HO-1 is inducible by numerous stimuli such as UV irradiation, heavy metals, endotoxin, cytokines, oxidants, and CO [2–4]. HO-1 plays a vital function in maintaining cellular homeostasis besides heme degradation. In the brain, the basal level of HO-1 expression is low, its expression increases under stress stimulation. Induction of HO-1 can effectively reverse neurodegenerative diseases such as stroke [5], Alzheimer's disease [6], Parkinson's disease [7], and multiple sclerosis [8]. However, in rat brain astrocyte- (RBA-) 1 cells, the neuroprotective effects of HO-1 induced by CO releasing molecule-3 (CORM-3) are not completely verified.

CO is a simple diatomic gas and long thought to be an environmental pollutant and a neurotoxin due to its high affinity for hemoglobin. CO arises in biological systems by the HO enzymes catabolizing heme. For many animal models of brain insults, low levels of CO administration, including CORMs and CO gas, could be a possible therapeutic strategy. For example, the low levels of inhaled CO decrease the infarct volume via an Nrf2 pathway in experimental middle cerebral artery occlusion (MCAO) models [9]. However, the clinical application of inhaled CO presents several disadvantages and limitations such as carboxyhemoglobin-related hypoxia. Thus, chemists developed prodrugs able to deliver CO, which were the first CORMs [10]. The water-soluble CORM-3 ([Ru(CO)_3_Cl(H_2_NCH_2_CO_2_)]) is one of the most studied prodrugs. CORM-3 could exert neuroprotection via reducing inflammatory responses [11]. Another report demonstrates that CORM-A1 diminishes the occurrence and clinical signs of the experimental allergic encephalomyelitis (EAE) and infiltrations of inflammatory cells in spinal cords [12]. However, the detailed mechanism underlying the CORM-3-mediated HO-1 expression is not completely defined in RBA-1 cells.

Several reports have demonstrated that CORMs stimulate ROS generation by NADPH oxidase (Nox) and mitochondria to induce the expression of antioxidant enzymes including HO-1 [13]. ROS exert as second messengers which trigger the HO-1 expression via modulating downstream signaling components and transcription factors [14–16]. Nox-dependent ROS generation could regulate the activities of mitogen-activated protein kinases (MAPKs) [17], phosphoinositide 3-kinase (PI3K)/Akt [18], mammalian target of rapamycin (mTOR), peroxisome proliferator-activated receptor (PPAR)*γ* [19], hypoxia-inducible factor 1 (HIF1) *α* [20], and ROS/nitric oxide (NO) production [21, 22]. Several redox-sensitive transcription factors, such as AP-1, NF-*κ*B, forkhead box O1 (FoxO1), and NF-E2-related factor 2 (Nrf2) bind with their binding sites in the regulatory elements of HO-1 gene promoter [23]. Especially, activation of FoxO1 and Nrf2 is well known as a cellular defender against oxidative stresses through the ARE-mediated expression of antioxidant genes such as HO-1 [24, 25]. However, the roles of the mTOR/FoxO1 pathway in CORM-3-induced HO-1 expression are still unknown in RBA-1 cells. The present study is aimed at examining the mechanisms of CORM-3-induced HO-1 expression in RBA-1 cells.

## 2. Material and Methods

### 2.1. Reagents and Antibodies

Dulbecco's modified Eagle's medium (DMEM)/F-12, fetal bovine serum (FBS), Lipofectamine 2000, OPTI-MEM, and siRNAs for p47 (RSS300253), Nox1 (RSS300165), Nox2 (RSS330363), Nox4 (RSS331680), p85 (RSS303756), Akt (RSS301983), and Nrf2 (RSS343557) were purchased from Invitrogen (Carlsbad, CA). Hybond-C membrane and enhanced chemiluminescence (ECL) western blotting detection systems were purchased from GE Healthcare Biosciences (Buckinghamshire, UK). Dimethyl sulfoxide (DMSO), tricarbonylchloro(glycinato)ruthenium (CORM-3), siRNAs for p110 (SASI_Rn02_00292737), FoxO1 (SASI_Rn02_00284211), and mTOR (SASI_Hs01_00203144), TRIzol, 2,3-bis-(2-methoxy-4-nitro-5-sulfophenyl)-2Htetrazolium-5-carbox-anilide (XTT) assay kit, and other chemicals were from Sigma (St. Louis, MO). Inactive form of CORM-3 (i-CORM-3) was prepared by dissolving CORM-3 in 0.01 mM phosphate-buffered saline (PBS) and CO was liberated at room temperature for 24 h. The bicinchoninic acid (BCA) protein assay reagents were from Thermo Scientific (Philadelphia, PA). Anti-phospho-Akt (Ser^473^), anti-phospho-FoxO1 (Ser^256^), anti-phospho-mTOR (Ser^2448^), anti-mTOR, and anti-p47^phox^ antibodies were from Cell Signaling (Danvers, MA). Anti-phospho-Nrf2 (Ser^40^), anti-Nox1, anti-Nox2, and anti-Nox4 antibodies were from Abcam (Cambridge, UK). Anti-glyceraldehyde-3-phosphate dehydrogenase (GAPDH) was from Encor (Gainesville, FL). Anti-lamin A, anti-p110, anti-p85, anti-Akt, anti-FoxO1, and anti-Nrf2 antibodies were from Santa Cruz (Santa Cruz, CA). Anti-HO-1 antibody, N-acetyl-L-cysteine (NAC), diphenyleneiodonium chloride (DPI), apocynin (APO), rotenone, LY294002, SH-5, rapamycin, and AS1842856 were from Enzo Life Sciences (Farmingdale, NY).

### 2.2. Cell Culture and Treatment

RBA-1 cells originated from neonatal rat cerebrum astrocytes and naturally developed through successive cell passages [26]. The use of the cell lines had been approved by Chang Gung University Institutional Animal Care and Use Committee (IACUC Approval No.: CGU16-081). The cells were cultured in DMEM/F-12 containing 5% FBS at cell density of 2 × 10^5^ cells/ml. Three days after the plating, 90% confluent cells were used for these experiments. and made quiescent at confluence by incubation in serum-free DMEM/F-12 for 24 h. Cells were incubated with CORM-3 or i-CORM-3 [27] at 37°C for the indicated time intervals, i-CORM-3 treatment had no effects on HO-1 induction, as compared with that of CORM-3 (Supplementary Figure 1). When inhibitors were used, cells were pretreated with the inhibitors for 1 or 2 h, as indicated before exposure to CORM-3, as previously described [28]. Treatment of RBA-1 cells with DMSO or the inhibitor alone had no significant effects on cell viability, as determined by an XTT assay kit (Supplementary Figure 2).

### 2.3. Protein Preparation and Western Blotting Analysis

RBA-1 cells were washed with ice-cold PBS and harvested in SDS-loading buffer (0.1 M Tris-HCl, pH 6.8; 1%SDS; 5% glycerol; 2.5% *β*-mercaptoethanol; and 0.02% bromophenol blue) to yield whole-cell extracts, as previously described [28]. Proteins were separated by SDS-PAGE and transferred by electrophoresis onto Hybond-C membranes. Membranes were incubated with antibodies at 1 : 1000 in Tween-Tris buffered saline (TTBS), and an anti-GAPDH antibody was used as an internal control. Membranes were washed with TTBS four times for 5 min and then incubated with 1 : 1500 secondary horseradish peroxidase-conjugated antibody for 1 h. Following washing, immunoreactive bands were detected by ECL and captured using a UVP BioSpectrum 500 Imaging System (Upland, CA). Image densitometry analyses were quantified using UN-SCAN-IT gel software (Rem, UT).

### 2.4. Total RNA Extraction and Real-Time Polymerase Chain Reaction (RT-PCR) Analysis

Quiescent RBA-1 cells were incubated with 30 *μ*M CORM-3 for 4 h in the presence or absence of the indicated inhibitors. Total RNA was extracted using TRIzol according to the manufacturer's protocol and was then reverse-transcribed to cDNA and analyzed by RT-PCR using a TaqMan gene expression assay system, as previously described [28], with sequences of primers and probes as follows:

HO-1: sense: 5′-TTTCACCTTCCCGAGCAT-3′, antisense: 5′-GCCTCTTCTGTCACCCTGT-3′, probe: 5′-CATGAACACTCTGGAGATGACC-3′; GAPDH: sense: 5′-AACTTTGGCATCGTGGAAGG-3′, anti-sense: 5′-GTGGATGCAGGGATGATGTTC-3′, probe: 5′-TGACCACAGTCCATGCCATCACTGC-3′.

RT-PCR was performed using a 7500 Real-Time PCR System (Applied Biosystems, Foster City, CA, USA). Relative gene expression was determined using the ^*ΔΔ*Ct^ method, with Ct indicating threshold cycles. All experiments were performed in triplicate.

### 2.5. Transient Transfection with Short Interfering RNA (siRNA)

At 70%–80% confluence, cells were transiently transfected with siRNAs (100 nM) corresponding to p47, Nox1, Nox2, Nox4, p110, p85, Akt, mTOR, FoxO1, Nrf2, or scrambled siRNA. GenMute™ reagent was used, followed by mixing with 75 *μ*l GenMute™ transfection buffer, as previously described [28]. After 10–15 min, 100 *μ*l of the mixture was applied directly to the cells. The cells were washed with PBS and maintained in DMEM/F-12 with 5% FBS for 24 h. Next, cells were starved in a serum-free DMEM/F-12 medium for 24 h. The transfection efficiency (approximate 60%) was determined by transfection with an enhanced green fluorescent protein (EGFP).

### 2.6. Plasmid Construction, Transfection, and Luciferase Reporter Gene Assays

A rat HO-1 promoter (accession no. J02722.1; −766 to +20) was constructed (sense: GGTACCCAGGAAGTCACAGTGTGGCC; antisense: CCCGAGCTCGTCG AGCTGTGGGCG CTCCAT) and cloned into the pGL3-basic vector containing a luciferase reporter system, as previously described [28]. To obtain ARE-luciferase reporter construct, double-stranded oligonucleotides containing a single copy of the 41-bp pair murine GSTYa ARE (5′-TAGCTTGGAAATGACATTGCTAATGGTG ACAAAGCAACTTT-3′; the core sequence underlined) were cloned into the pGL3 promoter vector (Promega, Madison, WI). All sequences of pARE-Luci were confirmed and verified the presence of the correct sequence and the absence of any other nucleotide changes by DNA sequencing. ARE-Luci activity was determined using a luciferase assay system, as previously described [14].

RBA-1 cells were transfected with plasmid DNA using Lipofectamine 2000. Co-transfection with pCMV-gal encoding for *β*-galactosidase was used as a control for transfection efficiency. To assess promoter activity, cells were collected and disrupted by sonication in a lysis buffer (25 mM Tris phosphate, pH 7.8, 2 mM ethylenediaminetetraacetic acid, 1% Triton X-100 and 10% glycerol). After centrifugation, aliquots of the supernatants were tested for luciferase activity using a luciferase assay system (Promega, Madison, WI). Firefly luciferase activities were standardized to *β*-galactosidase activity.

ARE: 5′-TAGCTTGGAAATGACATTGCTAATGGTGACAAAGCAACTTT-3′(sense), 5′-AAAGTTGCTTTGTCACCATTAGCAATGTCATTTCCAAGCTA-3′(sense), 5′-CTAGCTTGGAAATGACATTGCTAATGGTGACAAAGCAACTTTC-3'′(Kpn sense), 5′-TCGAGAAAGTTGCTTTGTCACCATTAGCAATGTCATTTCCAAGCTAGGTAC-3′(Xho antisense).

### 2.7. Isolation of Subcellular Fractions

RBA-1 cells were seeded in 10-cm dishes and starved for 24 h in serum-free DMEM/F-12 medium. After incubation with CORM-3, the cells were washed once with ice-cold PBS. 200 *μ*l of homogenization buffer A (20 mM Tris-HCl, pH 8.0, 10 mM EGTA, 2 mM EDTA, 2 mM dithiothreitol, 1 mM phenylmethylsulfonyl fluoride, 25 *μ*g/ml aprotinin, and 10 *μ*g/ml leupeptin) was added to each dish, and the cells were scraped into a 1.5 ml tube. The cytosolic and nuclear fractions were prepared by centrifugation, as previously described [14]. The protein concentration of each sample was determined by the BCA reagents. Samples from these supernatant fractions (30 *μ*g protein) were denatured and then subjected to SDS-PAGE using a 12% (*w*/*v*) running gel and transferred to nitrocellulose membrane. The levels of Nrf2, phospho-Nrf2, and lamin A in the nuclear fraction were determined by western blotting using an anti-Nrf2, anti-phospho-Nrf2, or lamin A antibody.

### 2.8. Measurement of Intracellular ROS Accumulation

Cells were cultured in DMEM/F-12 for 24 h and then treated with CORM-3. When inhibitors were used, they were added 1 h before the application of CORM-3. After washing twice with warm PBS, the cells were incubated with H_2_DCFDA (10 *μ*M) or DHE (5 *μ*M) for 30 min or 10 min, as previously described [29]. For ELISA assay, the fluorescence for DCF staining was detected at 495/529 nm, using a fluorescence microplate reader (Synergy^H1^ Hybrid Reader, BioTek). For immunofluorescence (IF) staining, washing thrice with cold-PBS, the images were observed under a fluorescence microscope (Axiovert 200 M, Zeiss).

### 2.9. Determination of NADPH Oxidase Activity Assay by Chemiluminescence Assay

After exposure to 30 *μ*M CORM-3 for the indicated time intervals, cells were gently scraped and centrifuged at 400 x g for 10 min at 4°C, as previously described [29]. The cell pellet was resuspended with 35 *μ*l of ice-cold PBS, and the cell suspension was kept on the ice. To a final 200 *μ*l volume of prewarmed (37°C) PBS containing either NADPH (1 *μ*M) or lucigenin (20 *μ*M), 5 *μ*l of cell suspension (2 × 10^4^ cells) was added to initiate the reaction followed by immediate measurement of chemiluminescence in a luminometer (Synergy^H1^ Hybrid Reader, BioTek).

### 2.10. Chromatin Immunoprecipitation Assay

To detect the association of the nuclear protein with rat HO-1 promoter, chromatin immunoprecipitation analysis was conducted, as previously described [29] with some modifications. Briefly, RBA-1 cells were crosslinked with 1% (*v*/*v*) formaldehyde at 37°C for 30 min and stop this reaction with 0.125 M glycine, then washed thrice with ice-cold PBS containing 1 mM PMSF, 1% (*v*/*v*) aprotinin, and 1% (*v*/*v*) leupeptin. Soluble chromatin was prepared using a ChIP assay kit (Upstate) according to the instructions of the manufacturer and immunoprecipitated without (control) or with an anti-Nrf2 antibody and normal goat immunoglobulin G (IgG). Following washing and elution, immunoprecipitates were heated overnight at 65°C to reverse crosslinking of DNA and protein. To avoid the possibility of amplification artifacts, PCR products for all SYBR Green primer pairs were verified to produce single products by agarose electrophoresis and a high-resolution melt curve. The relative mRNA levels were calculated using the comparative Ct method (^*ΔΔ*Ct^). The DNA was extracted and resuspended in H_2_O and subjected to PCR amplification with the ARE primers:

- ARE1: forward, 5′-ACAGTG TGGCCCAGGTTCTA-3′, reverse, 5′-TTCTAGCTGT GAGATGCTGGT-3′
- ARE2: forward, 5′- CTGGAGAATCTCAGGATTAAC-3′, reverse, 5′- ACCCTGTCTGGAAAAGACAA-3′

DNA (2 *μ*l) was extracted and resuspended in ddH_2_O (5 *μ*l), DMSO (1 *μ*l), and 2x Screening Fire Taq Master Mix (10 *μ*l) subjected to PCR amplification with the above primers ARE1 and ARE2. The amount of DNA-bound Nrf2 was expressed as a PCR product analyzed on 2% agarose 1× TAE gel containing ethidium bromide.

### 2.11. Immunofluorescent Staining

Growth-arrested cells were incubated with CORM-3 (30 *μ*M) for the indicated time intervals with or without APO, DPI, NAC, or rotenone pretreatment for 1 h. These cells were fixed, permeabilized, stained using anti-p-Nrf2 antibodies (1 : 200 dilutions) and 4′,6-diamidino-2-phenylindole (DAPI) after washing with ice-cold PBS, and finally mounted, as previously described [14]. The images of p-Nrf2 and nucleus were detected with a fluorescence microscope (Zeiss, Axiovert 200 M).

### 2.12. Data and Statistical Analysis

GraphPad Prizm Program 6.0 software (GraphPad, San Diego, CA) was adopted to perform statistical analysis. We used one-way ANOVA followed by Dunnett's post hoc test or nonparametric Kruskal–Wallis test followed by Dunn's post hoc test when comparing multiple independent groups. *P* values less than 0.05 were statistically significant. Only if *F* achieved *P* < 0.05 and the assumption of homogeneity of variance was also achieved, post hoc tests were run. All experiments were performed at least three individual times (*n* = number of independent cell culture preparations). All the data were expressed as the mean ± SEM. Error bars were omitted when they fell within the dimensions of the symbols.

## 3. Results

### 3.1. ROS Generation Is Involved in CORM-3-Induced HO-1 Expression

**Figure 1:**
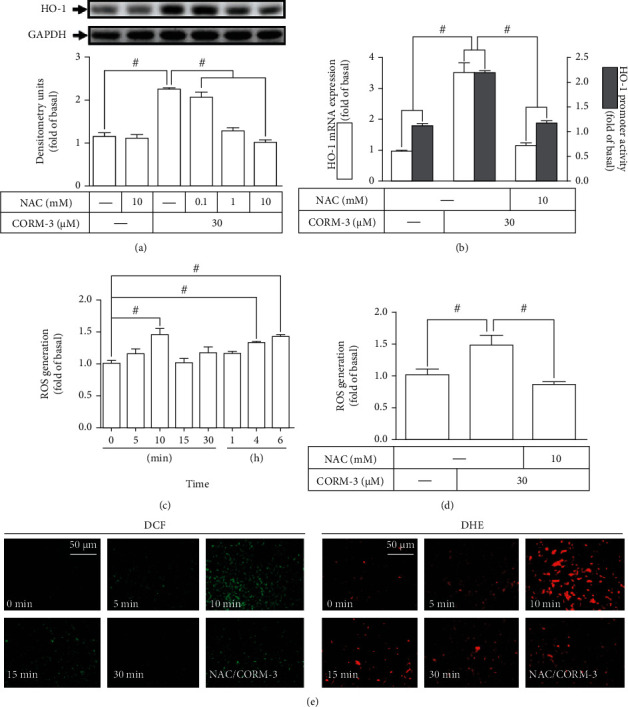
ROS generation involved in CORM-3-induced HO-1 expression. (a) RBA-1 cells were pretreated with various concentrations of NAC for 1 h and then incubated with 30 *μ*M CORM-3 for 6 h. The levels of HO-1 and GAPDH (as an internal control) were determined by western blot. (b) The cells were pretreated with NAC (10 mM) for 1 h and then incubated with CORM-3 (30 *μ*M) for 4 h. The levels of HO-1 and GAPDH mRNA were analyzed by real-time PCR (open bars). The cells were transiently transfected with HO-1 report gene together with a *β*-galactosidase plasmid, pretreated with NAC (10 mM) for 1 h, and then incubated with CORM-3 for 1 h. Promoter activity was determined in the cell lysates (solid bars). (c, d) The cells were pretreated with or without NAC (10 mM) for 1 h and then incubated with 30 *μ*M CORM-3 for the indicated time intervals (c) or 10 min (d). ROS generation was determined by measuring fluorescence intensity of DCF-DA. (e) The cells were pretreated with NAC (10 mM) for 1 h, incubated with 30 *μ*M CORM-3 for the indicated time intervals (CORM-3: 0, 5, 10, 15, and 30 min; NAC/CORM-3: 10 min), and then labeled with DCF-DA and DHE, respectively. The fluorescence intensity was observed under a fluorescence microscope. Data were expressed as mean ± S.E.M. of three independent experiments. Scale bar = 50 *μ*m. ^#^*P* < 0.05, as compared with the control or pretreatment with inhibitor indicated in the figure.

ROS are important factors in many physiological and pathological processes. Intracellular ROS have been shown to induce HO-1 gene expression [30]. To investigate whether ROS are involved in the CORM-3-induced HO-1 expression in RBA-1 cells, NAC was used for this purpose. Pretreatment with NAC inhibited the CORM-3-induced HO-1 protein expression in a concentration-dependent manner (Figure 1(a)). NAC pretreatment also reduced the CORM-3-induced HO-1 mRNA expression and promoter activity, determined by real-time PCR and promoter-luciferase assay, respectively (Figure 1(b)). To ensure that the generation of ROS plays a role in the HO-1 expression induced by CORM-3, as shown in Figure 1(c), 30 *μ*M CORM-3 time dependently induced ROS generation with an initial peak production within 10 min and followed by a second peak within 6 h. The production of ROS was reduced by NAC (Figure 1(d)). These results were further supported by using H_2_DCF-DA or DHE staining observed under a fluorescent microscope (Figure 1(e)). These results suggested that CORM-3 induces HO-1 expression via ROS generation in RBA-1 cells.

### 3.2. CORM-3-Induced HO-1 Expression Is Mediated via NADPH Oxidase

**Figure 2:**
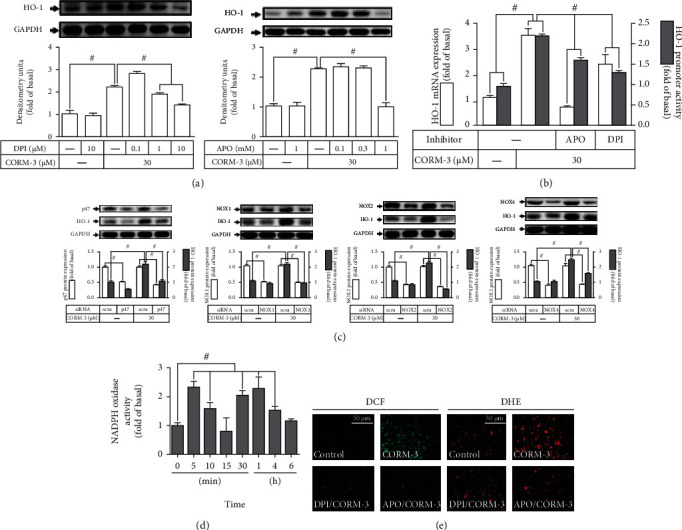
CORM-3-induced HO-1 expression is mediated via NADPH oxidase in RBA-1 cells. (a) The cells were pretreated with various concentrations of APO or DPI for 1 h and then incubated with 30 *μ*M CORM-3 for 6 h. The levels of HO-1 and GAPDH (as an internal control) protein expression were determined by western blot. (b) The cells were pretreated with APO (1 mM) or DPI (10 *μ*M) for 1 h and then incubated with 30 *μ*M CORM-3 for 4 h. The levels of HO-1 and GAPDH mRNA were determined by real-time PCR (open bars). The cells were transiently transfected with HO-1 report gene together with a *β*-galactosidase plasmid, subsequently pretreated with APO (1 mM) or DPI (10 *μ*M) for 1 h, and then incubated with 30 *μ*M CORM-3 for 1 h. Promoter activity was determined in the cell lysates (solid bars). (c) The cells were, respectively, transfected with p47, Nox1, Nox2, or Nox4 siRNA and then incubated with 30 *μ*M CORM-3 for 6 h. The levels of HO-1, p47, Nox1, Nox2, Nox4, and GAPDH (as an internal control) protein expressions were determined by western blot. (d) The cells were incubated with 30 *μ*M CORM-3 for the indicated time intervals. NADPH oxidase activity was determined by an ELISA assay kit. (e) The cells were pretreated with either APO (1 mM) or DPI (10 *μ*M) for 1 h, incubated with 30 *μ*M CORM-3 for 10 min, and then labeled with H_2_DCF-DA and DHE, respectively. The fluorescence intensity was observed under a fluorescence microscope. Scale bar = 50 *μ*m. Data were expressed as mean ± S.E.M. of three independent experiments. ^#^*P* < 0.05, as compared with the control, pretreatment with inhibitor, or siRNA indicated in the figure.

ROS have been shown to participate in HO-1 expression induced by CORM-3. Thus, we explored whether Nox is involved in the CORM-3-induced HO-1 expression, RBA-1 cells were pretreated with either DPI or APO (Nox inhibitors) for 2 h before exposure to 30 *μ*M CORM-3 for 6 h. As demonstrated in Figure 2(a), CORM-3-induced HO-1 protein expression was concentration dependently inhibited by pretreatment with either DPI or APO. CORM-3-induced HO-1 mRNA level and promoter activity were also attenuated by these inhibitors (Figure 2(b)). To uncover which isoform of Nox is involved in CORM-3-induced HO-1 expression, RBA-1 were transfected with p47, Nox1, Nox2, Nox4, or scrambled siRNA. As shown in Figure 2(c), downregulation of p47, Nox1, Nox2, or Nox4 protein levels attenuated the CORM-3-induced HO-1 expression. Besides, 30 *μ*M CORM-3 stimulated NOX activity with an initial peak within 5 min, followed by a second peak within 1 h, and declined close to the basal level within 6 h (Figure 2(d)**)**. We further ensured these results using H_2_DCF-DA and DHE staining. As illustrated in Figure 2(e), CORM-3-stimulated ROS accumulation was attenuated by either DPI or APO. These findings suggested that the HO-1 expression induced by CORM-3 is mediated through p47, Nox1, Nox2, or Nox4 activation in RBA-1 cells.

### 3.3. CORM-3-Induced HO-1 Expression Is Mediated via Mitochondrial Respiratory Complex

**Figure 3:**
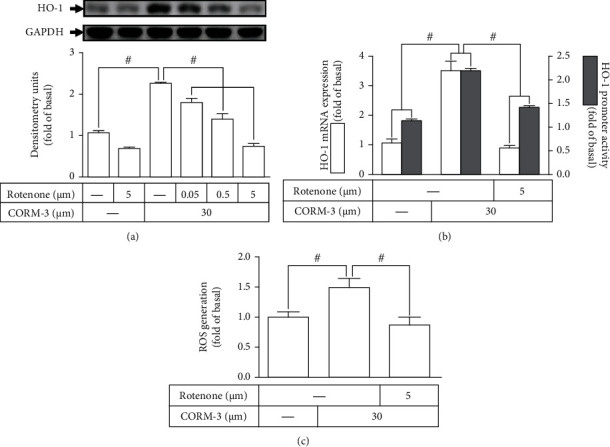
CORM-3-induced HO-1 expression is mediated via mitochondrial respiratory complex. (a) RBA-1 cells were pretreated with various concentrations of rotenone for 1 h and then incubated with 30 *μ*M CORM-3 for 6 h. The levels of HO-1 and GAPDH (as an internal control) protein expressions were determined by western blot. (b) The cells were pretreated with 5 *μ*M Rotenone for 1 h and then incubated with 30 *μ*M CORM-3 for 4 h. The levels of HO-1 and GAPDH mRNA were determined by real-time PCR (open bars). The cells were transiently transfected with HO-1 report gene together with a *β*-galactosidase plasmid, subsequently pretreated with 5 *μ*M Rotenone for 1 h, and then incubated with 30 *μ*M CORM-3 for 1 h. Promoter activity was determined in the cell lysates (solid bars). (c) The cells were pretreated with rotenone (5 *μ*M) for 1 h, incubated with 30 *μ*M CORM-3 for 10 min, and then labeled with H2DCF-DA. The fluorescence of DCF staining was detected using an ELISA assay. Data were expressed as mean ± S.E.M. of three independent experiments. ^#^*P* < 0.05, as compared with the control or pretreatment with inhibitor indicated in the figure.

The main sites of •O_2_^−^ formation are the flavin mononucleotide (FMN) site of complex I and the Q cycle of complex III in the mitochondrial respiratory complex [31]. However, the role of mitochondria-driven ROS in the CORM-3-induced HO-1 expression in RBA-1 cells was not understood. The cells were pretreated with rotenone (a mitochondrial complex inhibitor) for 2 h before exposure to 30 *μ*M CORM-3 for 6 h. Rotenone concentration dependently inhibited the HO-1 protein expression induced by CORM-3 (Figure 3(a)). Moreover, the HO-1 mRNA expression and promoter activity induced by CORM-3 were also attenuated by pretreatment with rotenone (Figure 3(b)**)**. To determine whether the production of ROS was mediated through activation of mitochondrial respiratory complex, CORM-3-stimulated mitochondrial ROS generation was blocked by pretreatment with rotenone (Figure 3(c)). These results implied that the HO-1 expression induced by CORM-3 is, at least partially, mediated through mitochondrial respiratory complex-driven ROS generation in RBA-1 cells.

### 3.4. CORM-3-Induced HO-1 Expression Is Mediated via PI3K/Akt Cascade

**Figure 4:**
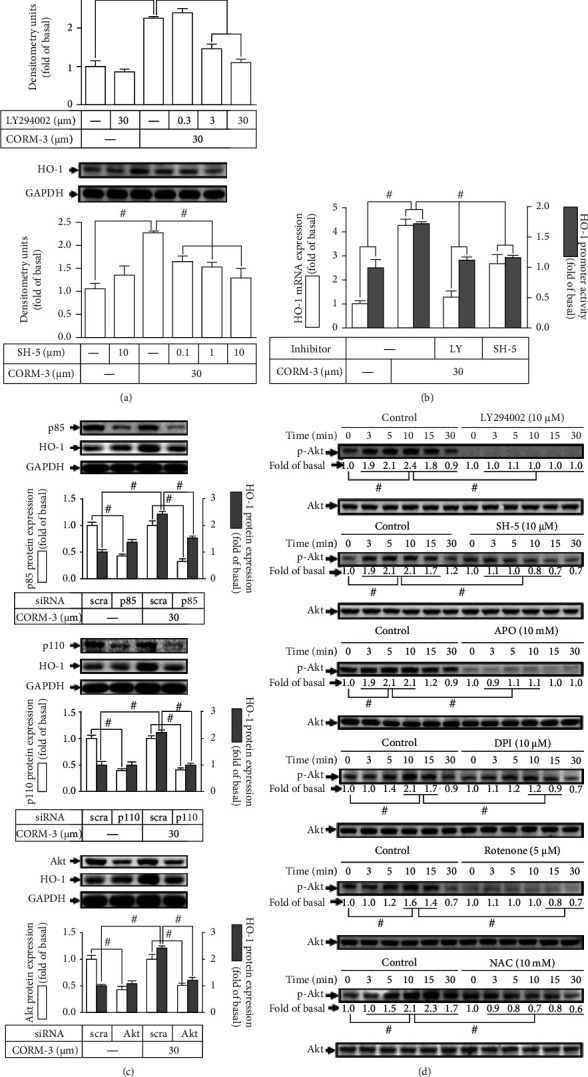
CORM-3-induced HO-1 expression is mediated via PI3K/Akt cascade. (a) RBA-1 cells were incubated with various concentrations of either LY294002 or SH-5 for 1 h and then incubated with 30 *μ*M CORM-3 for 6 h. The levels of HO-1 and GAPDH (as an internal control) protein expressions were determined by western blot. (b) The cells were pretreated with SH-5 (10 *μ*M) or LY294002 (10 *μ*M) for 1 h and then incubated with 30 *μ*M CORM-3 for 4 h. The levels of HO-1 and GAPDH mRNA were determined by real-time PCR (open bars). The cells were transiently transfected with HO-1 report gene together with a *β*-galactosidase plasmid, subsequently pretreated with SH-5 (10 *μ*M) or LY294002 (10 *μ*M) for 1 h, and then incubated with 30 *μ*M CORM-3 for 1 h. Promoter activity was determined in the cell lysates (solid bars). (c) The cells were transfected with Akt, p110, or p85 siRNA and then challenged with 30 *μ*M CORM-3 for 6 h. The protein levels of HO-1, Akt, p110, p85, and GAPDH (as an internal control) were determined by western blot. (d) The cells were pretreated without or with SH-5 (10 *μ*M), LY294002 (10 *μ*M), NAC (10 mM), APO (10 mM), DPI (10 *μ*M), or rotenone (5 *μ*M) for 1 h and then incubated with 30 *μ*M CORM-3 for the indicated time intervals. The levels of phosphorylated Akt and total Akt protein were determined by western blot. Data are expressed as the mean ± S.E.M. of three independent experiments. ^#^*P* < 0.05, as compared with the control, pretreatment with inhibitor, or siRNA indicated in the figure.

Several studies have revealed that the PI3K/Akt signaling pathway could trigger HO-1 upregulation in various types of cells [14, 32]. To investigate whether PI3K/Akt participated in HO-1 expression, the inhibitors of PI3K (LY294002) and Akt (SH-5) were used for these purposes. We found that pretreatment of RBA-1 cells with either LY294002 or SH-5 concentration dependently attenuated the HO-1 protein expression induced by CORM-3 (Figure 4(a)). Additionally, either LY294002 or SH-5 pretreatment also significantly reduced the HO-1 mRNA expression and promoter activity induced by CORM-3 (Figure 4(b)). To ensure the roles of PI3K/Akt in the CORM-3-induced HO-1 expression, cells were transfected with p110, p85, or Akt siRNA. Data in Figure 4(c) showed that knockdown of p110, p85, or Akt protein levels abolished the CORM-3-induced HO-1 expression. To prove whether CORM-3-stimulated PI3K/Akt activation was necessary for the HO-1 expression, the level of Akt phosphorylation was determined. Our data showed that CORM-3 time dependently stimulated Akt phosphorylation, which was attenuated by either SH-5 or LY294002 (Figure 4(d)). Moreover, pretreatment with NAC, APO, DPI, or rotenone also inhibited CORM-3-stimulated Akt phosphorylation (Figure 4(d)). These results suggested that in RBA-1, PI3K/Akt is involved in the HO-1 expression induced by CORM-3 and regulated by Nox- or mitochondrion-derived ROS signaling pathways.

### 3.5. CORM-3-Induced HO-1 Expression Is Mediated via mTOR

**Figure 5:**
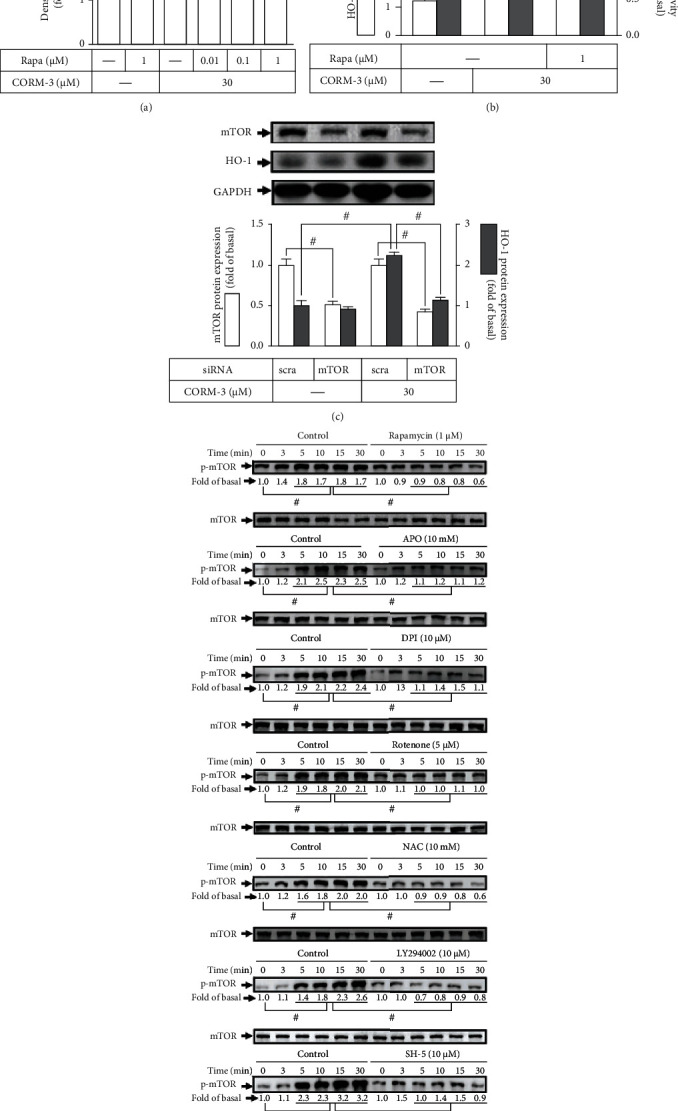
CORM-3-induced HO-1 expression is mediated via mTOR. (a) RBA-1 cells were incubated with various concentrations of rapamycin for 1 h and then incubated with 30 *μ*M CORM-3 for 6 h. The levels of HO-1 and GAPDH (as an internal control) protein expressions were determined by western blot. (b) The cells were pretreated with 1 *μ*M rapamycin for 1 h and then incubated with 30 *μ*M CORM-3 for 4 h. The levels of HO-1 and GAPDH mRNA were determined by real-time PCR (open bars). The cells were transiently transfected with HO-1 report gene together with a *β*-galactosidase plasmid, subsequently pretreated with 1 *μ*M rapamycin for 1 h, and then incubated with 30 *μ*M CORM-3 for 1 h. Promoter activity was determined in the cell lysates (solid bars). (c) The cells were transfected with mTOR siRNA and then challenged with 30 *μ*M CORM-3 for 6 h. The protein levels of HO-1, mTOR, and GAPDH (as an internal control) were determined by western blot. (d) The cells were pretreated with rapamycin (1 *μ*M), NAC (10 mM), APO (10 mM), DPI (10 *μ*M), rotenone (5 *μ*M), LY294002 (10 *μ*M), or SH-5 (10 *μ*M) for 1 h and then stimulated by 30 *μ*M CORM-3 for the indicated time intervals. The levels of phosphorylated mTOR and total mTOR proteins were determined by western blot. Data are expressed as the mean ± S.E.M. of three independent experiments. ^#^*P* < 0.05, as compared with the control, pretreatment with inhibitor, or siRNA indicated in the figure.

mTOR plays a critical role in diverse cellular functions including the expression of antioxidant enzymes such as HO-1 [33, 34]. To investigate whether mTOR is involved in the CORM-3-induced HO-1 expression, pretreatment with rapamycin significantly inhibited the HO-1 protein expression induced by CORM-3 (Figure 5(a)). Rapamycin also attenuated the HO-1 mRNA expression and promoter activity induced by CORM-3 (Figure 5(b)). To ensure the role of mTOR in HO-1 expression, the cells were transfected with mTOR siRNA to downregulate the mTOR protein level which attenuated the CORM-3-induced HO-1 expression (Figure 5(c)). We investigated whether mTOR activation was necessary for the HO-1 expression induced by CORM-3, and the level of mTOR phosphorylation was determined. CORM-3-stimulated mTOR phosphorylation was attenuated by rapamycin, APO, DPI, rotenone, NAC, LY294002, or SH-5 (Figure 5(d)). These results suggested that CORM-3-induced HO-1 expression is dependent on mTOR activation mediated through a Nox/mitochondria complex/ROS/PI3K/Ak cascade in RBA-1 cells.

### 3.6. FoxO1 Is Involved in CORM-3-Induced HO-1 Expression

**Figure 6:**
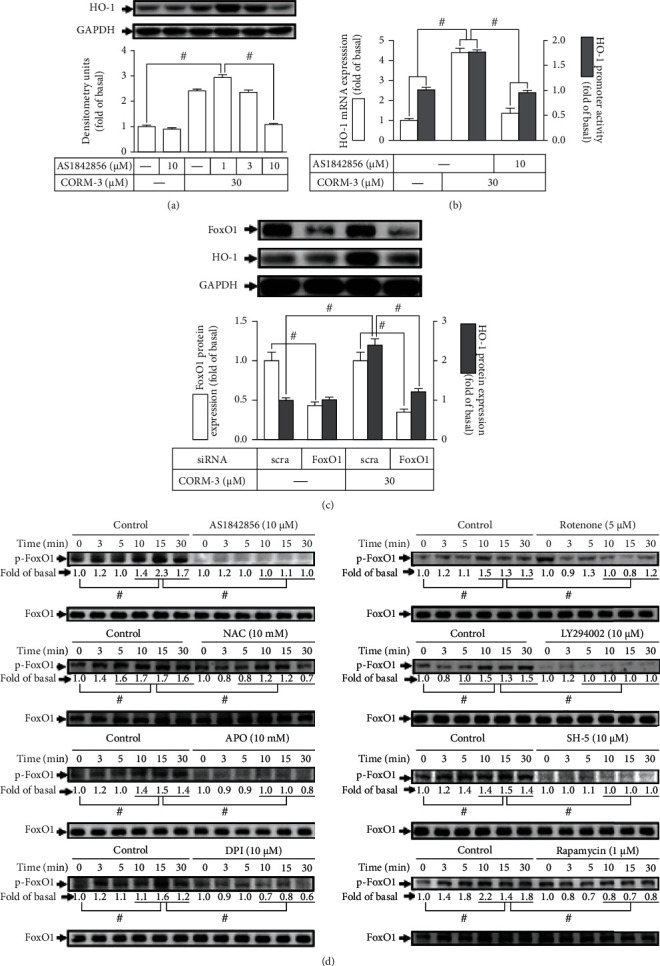
FoxO1 is involved in CORM-3-induced HO-1 expression. (a) RBA-1 cells were incubated with various concentrations of AS1842856 for 1 h and then incubated with 30 *μ*M CORM-3 for 6 h. The levels of HO-1 and GAPDH (as an internal control) protein expressions were determined by western blot. (b) The cells were pretreated with 10 *μ*M AS1842856 for 1 h and then incubated with 30 *μ*M CORM-3 for 4 h. The levels of HO-1 and GAPDH mRNA were determined by real-time PCR (open bars). The cells were transiently transfected with HO-1 report gene together with a *β*-galactosidase plasmid, subsequently pretreated with 10 *μ*M AS1842856 for 1 h, and then incubated with 30 *μ*M CORM-3 for 1 h. Promoter activity was determined in the cell lysates (solid bars). (c) The cells were transfected with FoxO1 siRNA and then challenged with 30 *μ*M CORM-3 for 6 h. The protein levels of HO-1, FoxO1, and GAPDH (as an internal control) were determined by western blot. (d) The cells were pretreated with AS1842856 (10 *μ*M), NAC (10 mM), APO (10 mM), DPI (10 *μ*M), rotenone (5 *μ*M), LY294002 (10 *μ*M), SH-5 (10 *μ*M), or rapamycin (1 *μ*M) for 1 h and then stimulated by 30 *μ*M CORM-3 for the indicated time intervals. The levels of phosphorylated FoxO1 and total FoxO1 protein were determined by western blot. Data are expressed as the mean ± S.E.M. of three independent experiments. ^#^*P* < 0.05, as compared with the control, pretreatment with inhibitor, or siRNA indicated in the figure.

*S*everal studies have shown that the activation of FoxO1 leads to the expression of many genes in various types of cells. However, whether FoxO1 activation is involved in the expression of HO-1 is not fully understood [35]. The pharmacological inhibitor of FoxO1 (AS1842856) was used to assess the role of FoxO1 in HO-1 expression. Pretreatment with AS1842856 concentration dependently attenuated the expression of HO-1 in RBA-1 cells stimulated by CORM-3 (Figure 6(a)). The HO-1 mRNA expression and promoter activity were also attenuated by AS1842856 in RBA-1 cells stimulated with CORM-3 (Figure 6(b)). To ensure the role of FoxO1 in HO-1 expression, FoxO1 was knocked down by transfection with FoxO1 siRNA, which downregulated the CORM-3-induced HO-1 expression (Figure 6(c)). To verify whether phosphorylation of FoxO1 was required for the HO-1 expression, the level of FoxO1 phosphorylation was examined. We found that CORM-3 time dependently stimulated FoxO1 phosphorylation which was attenuated by AS1842856 (Figure 6(d)). To differentiate whether CORM-3-stimulated FoxO1 phosphorylation was mediated via the Nox/mitochondria complex/ROS/PI3K/Akt/mTOR cascade, as shown in Figure 6(d), the level of FoxO1 phosphorylation stimulated by CORM-3 was inhibited by NAC, APO, DPI, rotenone, LY294002, SH-5, or rapamycin. These findings suggested that a Nox/mitochondria/ROS/PI3K/Akt/mTOR-dependent FoxO1 activation is involved in HO-1 expression induced by CORM-3 in RBA-1 cells.

### 3.7. Nrf2 Contributes to CORM-3-Induced HO-1 Expression

**Figure 7:**
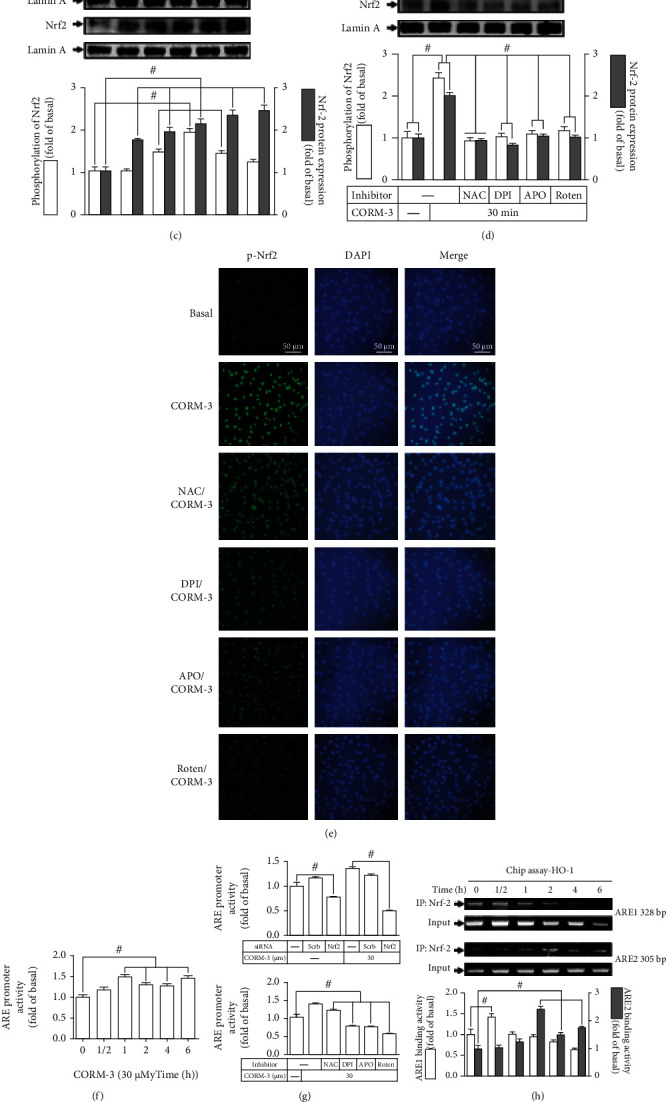
Nrf2 contributes to CORM-3-induced HO-1 expression. (a) RBA-1 cells were transfected with Nrf2 siRNA and then challenged with 30 *μ*M CORM-3 for 6 h. The protein levels of HO-1, Nrf2, and GAPDH were determined by western blot. (b) The cells were transfected with Nrf2 siRNA and then incubated with 30 *μ*M CORM-3 for 4 h. The levels of HO-1 and GAPDH mRNA were determined by real-time PCR (open bars). The cells were transiently transfected with HO-1 report gene together with a *β*-galactosidase plasmid, followed by transfected with Nrf2 siRNA, and then incubated with 30 *μ*M CORM-3 for 1 h. Promoter activity was determined in the cell lysates (solid bars). (c) The cells were challenged with 30 *μ*M CORM-3 for the indicated time intervals. The cell lysates were centrifuged to prepare nuclear fraction. The levels of Nrf2, phosphorylated-Nrf2, and lamin A were determined by western blot. (d) The cells were pretreated with NAC (10 mM), DPI (10 *μ*M), APO (10 mM), or rotenone (5 *μ*M) for 1 h and then incubated with 30 *μ*M CORM-3 for 30 min. The nuclear fraction was prepared and analyzed by western blot. (e) Cells were pretreated with or without rotenone (5 *μ*M), NAC (10 mM), APO (10 mM), or DPI (10 *μ*M) for 1 h and then stimulated by CORM-3 for 30 min. These cells were stained using anti-p-Nrf2 antibodies and DAPI. The images of p-Nrf2 and nucleus were detected with a fluorescence microscope. Scale bar = 50 *μ*m. (f) The cells were transiently transfected with ARE report gene together with a *β*-galactosidase plasmid and then incubated with 30 *μ*M CORM-3 for the indicated time intervals. ARE promoter activity was determined by a luciferase reporter gene assay. (g) The cells were transfected with Nrf2 siRNA, followed by transiently transfected with ARE report gene together with a *β*-galactosidase plasmid, and then challenged with 30 *μ*M CORM-3 for 1 h (upper panel). The cells transfected with ARE report gene together with a *β*-galactosidase plasmid were pretreated with NAC (10 mM), APO (10 mM), DPI (10 *μ*M), or rotenone (5 *μ*M) for 1 h and then stimulated by 30 *μ*M CORM-3 for 1 h (lower panel). The levels of ARE promoter activity were determined in the cell lysates. (h, i) Cells were treated with 30 *μ*M CORM-3 for the indicated time points (h) or pretreated with rotenone (5 *μ*M), NAC (10 mM), APO (10 mM), or DPI (10 *μ*M) for 1 h and then stimulated by CORM-3 for 2 h (i). The levels of Nrf2 binding to ARE region of the HO-1 promoter were detected by a ChIP assay. Data are expressed as the mean ± S.E.M. of three independent experiments. ^#^*P* < 0.05, as compared with the control, pretreatment with inhibitor, or siRNA indicated in the figure. DAPI: 4′,6-diamidino-2-phenylindole.

It has been shown that activation of Nrf2 by external stimuli is a key player in the expression of HO-1 in various types of cells [36]. Some studies have revealed that CORMs activate the Nrf2/HO-1 axis to protect against the inflammatory responses under various pathological conditions [9, 37]. However, in RBA-1 cells, the involvement of Nrf2 in CORM-3-induced HO-1 expression was not completely defined. To evaluate the role of Nrf2 in HO-1 expression, Nrf2 protein expression was downregulated by transfection with Nrf2 siRNA, which abrogated the HO-1 expression induced by CORM-3 (Figure 7(a)). Transfection with Nrf2 siRNA also attenuated CORM-3-induced HO-1 mRNA expression and promoter activity (Figure 7(b)). To evaluate the function of Nrf2 in HO-1 expression, CORM-3 stimulated Nrf2 phosphorylation and translocation into the nuclear fraction (Figure 7(c)) which were attenuated by pretreatment with NAC, DPI, APO, or rotenone (Figure 7(d)). These results were further supported by the data of immunofluorescent staining to verify the role of Nrf2 in the CORM-3-mediated responses in RBA-1 cells (Figure 7(e)). These findings implied that activation of Nrf2 was mediated through a Nox/mitochondrial complex/ROS pathway.

Nrf2 can activate the antioxidant response element (ARE), leading to transcriptional activation of antioxidant genes. To determine whether ARE-driven transcriptional activity is involved in the HO-1 expression induced by CORM-3, data of ARE promoter-luciferase assay showed that CORM-3 time dependently enhanced ARE transcriptional activity which reached a maximal response within 1 h (Figure 7(f)). Moreover, transfection with Nrf2 siRNA and pretreatment with NAC, DPI, APO, or rotenone attenuated CORM-3-stimulated ARE promoter activity (Figure 7(g)). Further, ChIP assay showed that CORM-3 enhanced the binding of Nrf2 to ARE2 on HO-1 promoter with a maximal response within 2 h, but not to ARE1 **(**Figure 7(h)). To further reveal whether ROS participate in Nrf2 recruitment to ARE2 promoter, pretreatment with rotenone, DPI, NAC, or APO attenuated the binding of Nrf2 to ARE2 on HO-1 promoters (Figure 7(i)). These results indicated that CORM-3 stimulated a Nox/mitochondrial complex/ROS-dependent Nrf2 activation, leading to transactivation of ARE2 and HO-1 transcription in RBA-1 cells.

## 4. Discussion

**Figure 8:**
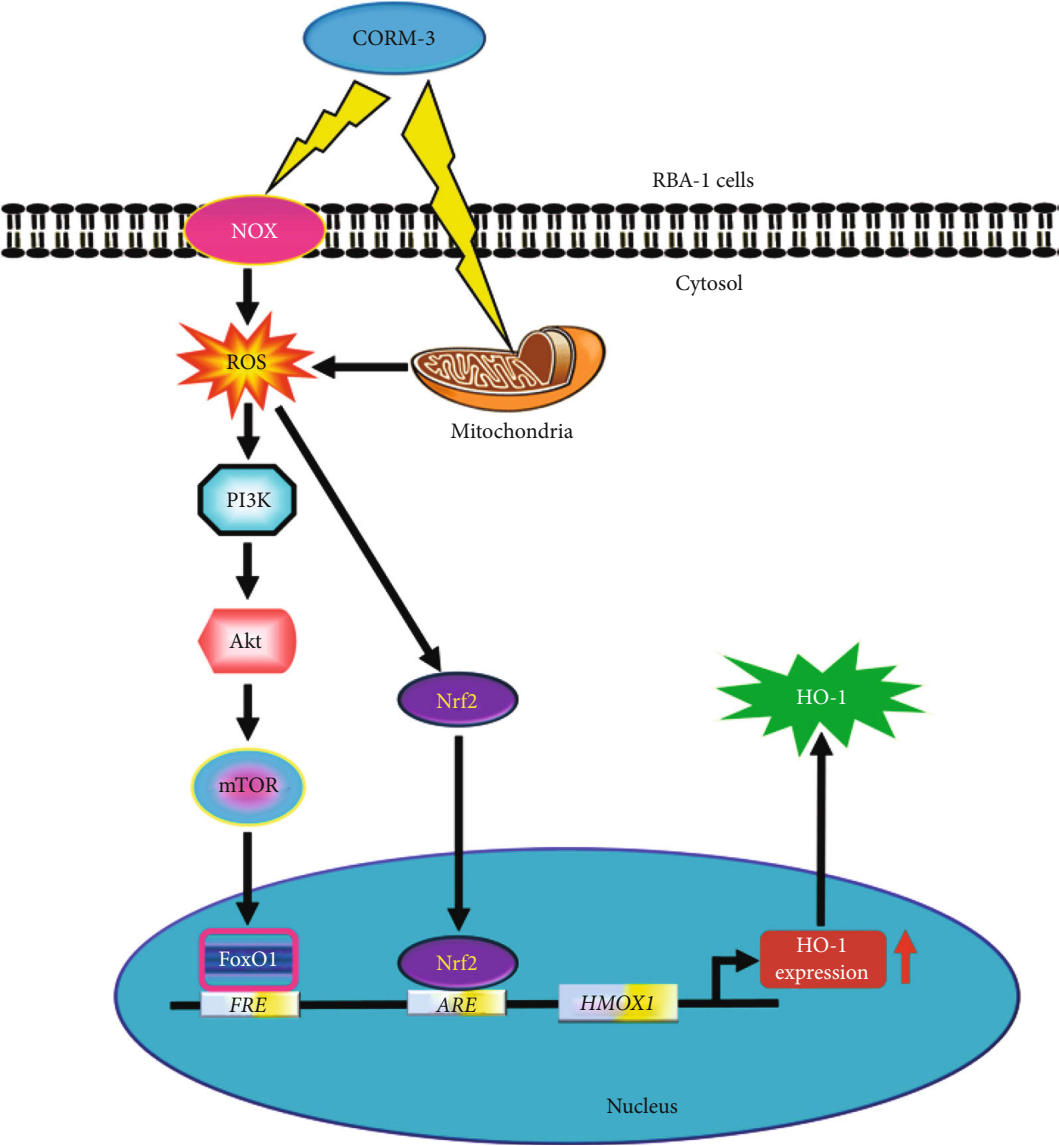
A schematic pathway for CORM-3-induced HO-1 expression in RBA-1 cells. CORM-3 enhanced NADPH oxidase and/or mitochondrial respiratory complex activity, which resulted in the accumulation of intracellular ROS. Oxidative stress promoted the phosphorylation of PI3K/Akt/mTOR and the activation of FoxO1 and Nrf2. After nuclear translocation, FoxO1 and Nrf2 bind to the ARE2 region of HO-1 promoter and increase the expression of the HO-1 gene in RBA-1 cells. Abbreviation: FRE: canonical forkhead response element; *HMOX1*: gene of HO-1.

CO/HO-1 is recognized as a cytoprotective factor under various pathophysiological situations [5, 7, 8, 38, 39]. CO can activate the Nox activity and ROS generation to induce HO-1 expression and possess the effects of anti-inflammation and antioxidation [40]. Our previous study has shown that CORM-3 sequentially activated the c-Src/Pyk2/PKC*α*/p42/p44 MAPK/AP-1 pathway, thereby upregulating HO-1 in RBA-1 cells [28]. The results of the present study further indicated that CORM-3-induced HO-1 gene expression was attenuated by ROS scavenger, Nox inhibitors (DPI and APO), and rotenone. Further, the downstream components of Nox/ROS were differentiated by the application of selective genetic silencing and pharmacological inhibitors. Our results showed that, in RBA-1, CORM-3-enhanced HO-1 expression is mediated through activation of Nox-mitochondria/ROS-dependent either PI3K/Akt/mTOR/FoxO1 or Nrf2 pathways (Figure 8). To the best of our knowledge, this study is the first to address that the ROS-mediated mTOR/FoxO1 pathway is involved in the CORM-3-induced HO-1 expression in RBA-1 cells.

CO can cause vasodilation through soluble guanylyl cyclase (sGC) and cyclic GMP (cGMP) [41]. Astrocytes have been supported to regulate the HO/CO pathway of cerebral vasodilation mediated through an increased intracellular Ca^2+^ in astrocytes themselves by activating the ionotropic glutamate receptors (iGluR) [42]. CO protects cerebral vascular endothelium from oxidative stress and apoptosis caused by proinflammatory mediators [43]. Thus, accumulating evidence has indicated that CO could act as a strategy for the management of ischemic and hemorrhagic stroke and multiple sclerosis, which is a major regulator of cerebrovascular hemodynamics and inflammatory reaction in the brain [44].

ROS function as second messengers that regulate intracellular signaling components associated with both inflammatory responses and normal physiological functions dependent on their levels [45, 46]. In astrocytes, Nox-dependent ROS generation is associated with the HO-1 expression induced by CORM-2 [14]. Thus, we investigated the causal relationship between Nox-derived ROS and HO-1 expression in RBA-1 challenged with CORM-3. NAC, a thiol-containing compound, possesses therapeutic effects on various psychiatric/neurological disorders, which are mediated through scavenging ROS and bind metal ions into complexes [47]. Both APO (a p47^phox^ inhibitor) and DPI (a Nox inhibitor) belong to Nox-related inhibitors have been shown to inhibit Nox activation by preventing p47^phox^ (a Nox subunit) translocation to the plasma membrane [48]. Our results showed that Nox/ROS plays an important role in HO-1 expression because CORM-3-induced ROS generation and HO-1 expression were inhibited by NAC, DPI, or APO. Nox is a multisubunit enzyme including membrane and cytosolic subunits. Nox family has seven members with various tissue distributions and activation mechanisms in many cells. According to our results, we revealed that Nox1, Nox2, and Nox4 are involved in ROS generation leading to the HO-1 expression induced by CORM-3. These results were supported by transfection with their respective siRNAs. The generation of mitochondrial ROS was also involved in the CORM-3-induced HO-1 expression, which was ensured by the downregulation of HO-1 expression by pretreatment with rotenone in RBA-1. CORM-3 can uncouple mitochondria and decrease the proton electromotive force, leading to reducing reverse electron flow and ROS production at the level of complex I [49]. In addition, previous studies have indicated that CORM-3/CO induces ROS generation by complex III due to inhibition of cytochrome *c* oxidase [49, 50]. These data are consistent with previous studies demonstrating that, in tracheal smooth muscle cells, Nox-derived ROS generation is involved in HO-1 induction [15, 16]. Our data suggested that Nox- and mitochondria-derived ROS are, at least partially, involved in the HO-1 expression induced by CORM-3 in RBA-1 cells.

The induction of HO-1 is regulated by Nox/ROS-dependent PI3K/Akt pathways [51]. Accumulating evidence has indicated that CO has cytoprotective effects on hepatic ischemia/reperfusion injury and enhancing neurotrophic factor expression via activating the PI3K/Akt pathway [22, 52]. In numerous cells, different inducers including CO can activate the PI3K/Akt pathway to mediate HO-1 gene expression [14, 32]. Our results demonstrated that PI3K/Akt phosphorylation is involved in the HO-1 expression stimulated by CORM-3. PI3K/Akt are also downstream targets of Nox and mitochondria-ROS generation since Akt phosphorylation stimulated by CORM-3 was attenuated by the inhibitor of either Nox or mitochondria. These results concluded that a Nox and mitochondria-ROS-dependent PI3K/Akt phosphorylation participates in the HO-1 expression induced by CORM-3.

PI3K/Akt or AMPK (AMP-activated protein kinase) which could regulate tuberous sclerosis complex (TSC) and Ras-homolog enriched in the brain (Rheb), which has emerged as a modulator of mTOR pathway to activate transcription and translation activities via phosphorylates 4E-BPs, which, in turn, stimulate eIF4E [53, 54]. Moreover, PI3K/Akt further activates transcription factors, such as FoxO [55]. mTOR integrates diverse signals leading to various cellular responses, including inflammation. PI3K/Akt inhibits the activity of TSCs by phosphorylating the TSC2 component [56], finally attenuating the inhibitory effect of the TSC complex toward mTOR complex (TORC)1 [57]. A recent study showed that salvianolic acid A-induced Nrf2/HO-1 activation and cytoprotection are mediated through Akt/mTORC1 activation [33]. Another study also revealed that melatonin activates an Akt/mTOR-dependent pathway to induce HO-1 expression, which prevents hemorrhagic shock-induced liver injury [34]. Our results indicated that CORM-3-induced HO-1 expression was attenuated by rapamycin or transfection with mTOR siRNA, compatible with these studies. We further demonstrated that mTOR is a downstream target of Nox and mitochondria-ROS generation/Akt cascade because CORM-3-stimulated mTOR phosphorylation was attenuated by NAC, rotenone, the inhibitors of Nox, LY294002, and SH-5. These results implied that a Nox and mitochondria-ROS/PI3K/Akt-dependent mTOR phosphorylation participates in the HO-1 expression induced by CORM-3 in RBA-1 cells.

FoxO members mainly comprise FoxO1, FoxO3, FoxO4, and FoxO6. These FoxO members specifically bind to conserved DNA sequence 5′-TTGTTTAC-3′ via their sharing conserved DNA-binding domains with a structure of helix-turn-helix [58]. FoxO regulates a series of downstream targets via transcriptional processes, thus involved in several pathophysiological activities [59]. Multiple signaling pathways converge on FoxOs, including PI3K/Akt [60]. Activated Akt can phosphorylate FoxO1 at three sites (Thr^24^, Ser^256^, and Ser^319^) which results in increased binding of FoxO1 to the regulator 14-3-3, leading to export FoxO1 from nucleus to cytoplasm [23]. Previous studies indicated that FoxO1 is involved in the HO-1 expression which has cytoprotecting effects in various injury models [24, 25]. Our findings are the first to demonstrate that HO-1 expression induced by CORM-3 was attenuated by FoxO1 inhibitor AS1842856 or transfection with its siRNA. We observed that pretreatment with AS1842856, NAC, APO, DPI, rotenone, LY294002, or SH-5 attenuated FoxO1 phosphorylation stimulated by CORM-3. These findings concluded that FoxO1 is downstream signaling of Nox and mitochondria-ROS/PI3K/Akt-dependent mTOR cascade leading to HO-1 expression in RBA-1 cells.

Our recent research has reported that the MAPK-dependent AP-1 pathway is an essential mechanism for the CORM-3-induced HO-1 expression in RAB-1 cells [28]. Moreover, several redox-sensitive transcription factors including Nrf2 have been shown to bind with their binding sites in the regulatory elements of the HO-1 gene promoter [23]. Nrf2 is well known as a cellular defender against oxidative stresses and electrophilic insults through the ARE-mediated expression of HO-1. Under physiological conditions, Nrf2 is bound with Keap1 and degraded through Keap1-mediated ubiquitination. Upon cells exposed to oxidative or chemical stress, Nrf2 is liberated from its inhibitor Keap1, then leading to nuclear translocation and binding with the ARE located on the promoters of Nrf2 target genes. Interestingly, Nrf2/HO-1 can also be upregulated by lipopolysaccharides under glutamine depletion in microglial cells [61]. Some experiments have shown that CORMs/CO possesses anti-inflammatory and neuroprotective effects through the Nrf2-dependent HO-1 expression in various types of cells [9, 37]. Our previous study also demonstrated that CORM-2/CO induced HO-1 expression through ROS-dependent Nrf2 pathway [14]. In this study, our results uncovered that Nrf2 activation regulates the HO-1 expression induced by CORM-3 because the response is attenuated by its siRNA transfection. We further differentiated the role of Nrf2 in the signaling pathway of HO-1 expression and demonstrated that the phosphorylation and nuclear translocation of Nrf2 were attenuated by NAC, DPI, APO, and rotenone. Moreover, ChIP assay revealed the interaction between Nrf2 and ARE on HO-1 promoter, showing that the binding site of Nrf2 located on the ARE2 region but not ARE1 region on the HO-1 promoter and the binding ability of Nrf2 with ARE2 region was attenuated by NAC, DPI, APO, and rotenone.

## 5. Conclusions

In summary, we found that the HO-1 expression induced by CORM-3 is partially mediated through Nox-mitochondria/ROS-dependent PI3K/Akt/mTOR/FoxO1 cascade and Nrf2 activity in RBA-1 cells. Therefore, CORM-3 might be a potential strategy for upregulation of HO-1 and management of brain inflammatory and degenerative diseases.

## Data Availability

The data used to support the findings of this study are available from the corresponding author upon request.

## Abbreviations

APO: Apocynin
ARE: Antioxidant response element
BSA: Bovine serum albumin
ChIP: Chromatin immunoprecipitation
CM-H_2_DCFDA: 5-(and-6)-Chloromethyl-2′,7′-dichlorodihydrofluorescein diacetate, acetyl ester
CO: Carbon monoxide
CORM: Carbon monoxide-releasing molecules
CORM-3: Tricarbonylchloro(glycinato)ruthenium
CREB: Cyclic adenosine 3,5-monophosphate-responsive element-binding protein
DHE: Dihydroethidium
DMEM: Dulbecco's modified Eagle's medium
DNA: Deoxyribonucleic acid
DMSO: Dimethyl sulfoxide
DPI: Diphenyleneiodonium chloride
EAE: Experimental allergic encephalomyelitis
ECL: Enhanced chemiluminescence
EGFP: Enhanced green fluorescent protein
FMN: Flavin mononucleotide
FBS: Fetal bovine serum
FoxO1: Forkhead box O1
GAPDH: Glyceraldehyde-3-phosphate dehydrogenase
HIF1: Hypoxia-inducible factor 1
HO: Heme oxygenase
MAPK: Mitogen-activated protein kinase
Keap1: Kelch ECH associating protein 1
MCAO: Middle cerebral artery occlusion
mTOR: Mammalian target of rapamycin
NAC: N-acetyl-L-cysteine
Nox: NADPH oxidase
Nrf2: NF-E2-related factor 2
NO: Nitric oxide
PBS: Phosphate-buffered saline
PI3K: Phosphoinositide 3-kinase
PPAR: Peroxisome proliferator-activated receptor
RBA: Rat brain astrocytes
RNA: Ribonucleic acid
ROS: Reactive oxygen species
RT-PCR: Reverse transcription-polymerase chain reaction
SDS-PAGE: Sodium dodecyl sulfate-polyacrylamide gel electrophoresis
SEMs: Standard errors of the mean
siRNA: Small interfering RNA
STAT: Signal transducers and activators of transcription
TTBS: Tween-Tris-buffered saline
XTT: 2,3-Bis-(2-methoxy-4-nitro-5-sulfophenyl)-2Htetrazolium-5-carbox-anilide.

## Supplementary Materials

Supplementary Materials Supplementary Figure 1: effects of either CORM-3 or inactive-CORM-3 on HO-1 expression on RBA-1 cells. Supplementary Figure 2: effects of CORM-3 on cell viability of RBA-1 cells.
