# Supplementary material for: Carbon Monoxide Releasing Molecule-3 Enhances Heme Oxygenase-1 Induction via ROS-Dependent FoxO1 and Nrf2 in Brain Astrocytes
Source: Oxid Med Cell Longev. 2021 Jun 12;2021:5521196. doi: 10.1155/2021/5521196 (PMC8214505; doi:10.1155/2021/5521196)
Supplement: Supplementary Materials — Supplementary Figure 1: effects of either CORM-3 or inactive-CORM-3 on HO-1 expression on RBA-1 cells. Supplementary Figure 2: effects of CORM-3 on cell viability of RBA-1 cells. [file 5521196.f1.pdf]

Supplementary Figure 1.

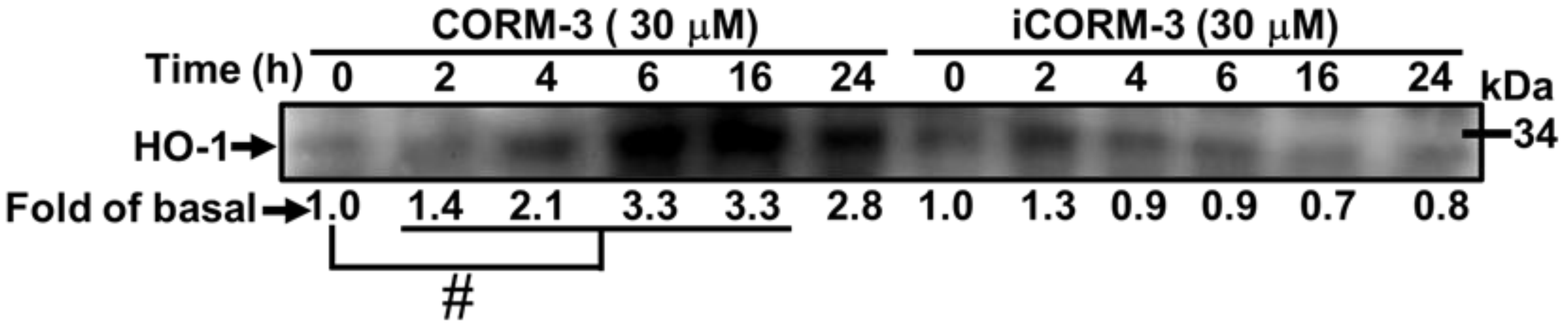

Supplementary Figure 1. RBA-1 cells were incubated with 30 μM either CORM-3 or i-CORM-3 for the indicated time intervals and the levels of HO-1 were determined by western blot. #p < 0.05, as compared with respective control.

Supplementary Figure 2.

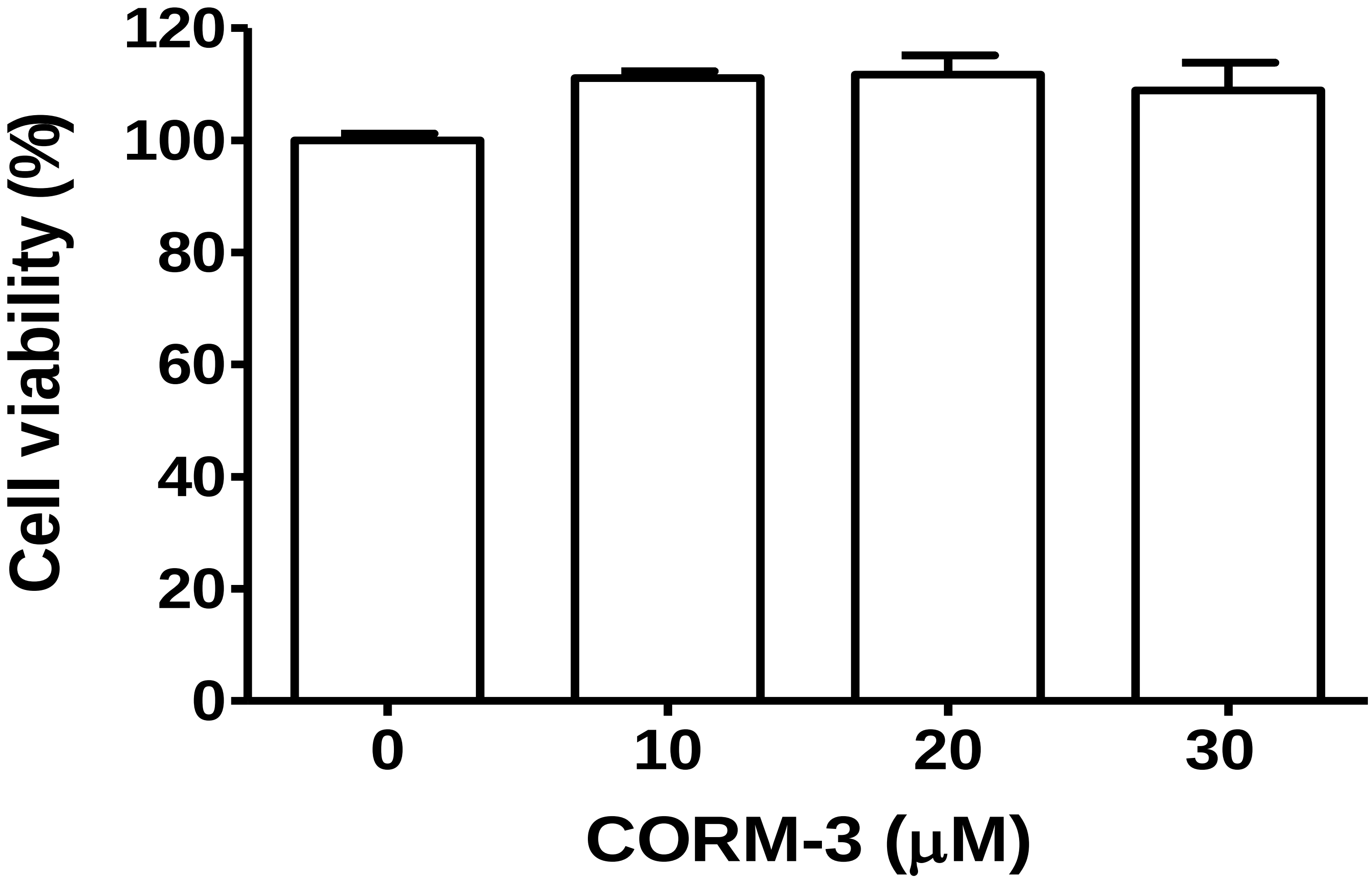

Supplementary Figure 2. RBA-1 cells were incubated with or without the indicated dosages (10, 20, 30 μM) of CORM-3 for 24 h and the levels of cell viability were determined by an XTT assay.
